# Hindbrain Administration of Oxytocin Reduces Food Intake, Weight Gain and Activates Catecholamine Neurons in the Hindbrain Nucleus of the Solitary Tract in Rats

**DOI:** 10.3390/jcm10215078

**Published:** 2021-10-29

**Authors:** Vishwanath T. Anekonda, Benjamin W. Thompson, Jacqueline M. Ho, Zachary S. Roberts, Melise M. Edwards, Ha K. Nguyen, Andrew D. Dodson, Tami Wolden-Hanson, Daniel W. Chukri, Adam J. Herbertson, James L. Graham, Peter J. Havel, Tomasz A. Wietecha, Kevin D. O’Brien, James E. Blevins

**Affiliations:** 1 VA Puget Sound Health Care System, Office of Research and Development Medical Research Service, Department of Veterans Affairs Medical Center, Seattle, WA 98108, USA; v.anekonda@gmail.com (V.T.A.); bthompson2@zagmail.gonzaga.edu (B.W.T.); jacks529@gmail.com (J.M.H.); zroberts318@gmail.com (Z.S.R.); medwards@umass.edu (M.M.E.); nguyha16@gmail.com (H.K.N.); anddodson@gmail.com (A.D.D.); tami.woldenhanson@gmail.com (T.W.-H.); chukrd@outlook.com (D.W.C.); adamherb12@gmail.com (A.J.H.); 2 Division of Metabolism, Endocrinology and Nutrition, Department of Medicine, University of Washington School of Medicine, Seattle, WA 98195, USA; wietechatomasz@gmail.com; 3 Department of Nutrition and Department of Molecular Biosciences, School of Veterinary Medicine, University of California, Davis, CA 95616, USA; jlgraham@ucdavis.edu (J.L.G.); pjhavel@ucdavis.edu (P.J.H.); 4 UW Medicine Diabetes Institute, University of Washington School of Medicine, Seattle, WA 98109, USA; cardiac@cardiology.washington.edu; 5 Division of Cardiology, Department of Medicine, University of Washington School of Medicine, Seattle, WA 98195, USA

**Keywords:** satiety, meal size, oxytocin, hindbrain

## Abstract

Existing studies show that CNS oxytocin (OT) signaling is important in the control of energy balance, but it is unclear which neurons may contribute to these effects. Our goals were to examine (1) the dose-response effects of acute OT administration into the third (3V; forebrain) and fourth (4V; hindbrain) ventricles to assess sensitivity to OT in forebrain and hindbrain sites, (2) the extent to which chronic 4V administration of OT reduces weight gain associated with the progression of diet-induced obesity, and (3) whether nucleus tractus solitarius (NTS) catecholamine neurons are downstream targets of 4V OT. Initially, we examined the dose-response effects of 3V and 4V OT (0.04, 0.2, 1, or 5 μg). 3V and 4V OT (5 μg) suppressed 0.5-h food intake by 71.7 ± 6.0% and 60 ± 12.9%, respectively. 4V OT (0.04, 0.2, 1 μg) reduced food intake by 30.9 ± 12.9, 42.1 ± 9.4, and 56.4 ± 9.0%, respectively, whereas 3V administration of OT (1 μg) was only effective at reducing 0.5-h food intake by 38.3 ± 10.9%. We subsequently found that chronic 4V OT infusion, as with chronic 3V infusion, reduced body weight gain (specific to fat mass) and tended to reduce plasma leptin in high-fat diet (HFD)-fed rats, in part, through a reduction in energy intake. Lastly, we determined that 4V OT increased the number of hindbrain caudal NTS Fos (+) neurons (156 ± 25) relative to vehicle (12 ± 3). The 4V OT also induced Fos in tyrosine hydroxylase (TH; marker of catecholamine neurons) (+) neurons (25 ± 7%) relative to vehicle (0.8 ± 0.3%). Collectively, these findings support the hypothesis that OT within the hindbrain is effective at reducing food intake, weight gain, and adiposity and that NTS catecholamine neurons in addition to non-catecholaminergic neurons are downstream targets of CNS OT.

## 1. Introduction

Existing studies suggest that central release of the nonapeptide, oxytocin (OT), which is expressed predominantly in the paraventricular nucleus (PVN) and supraoptic nucleus (SON), plays an important role in the regulation of energy balance [1]. Past studies have demonstrated that OT reduces energy intake, body weight and/or weight gain in diet-induced obese (DIO) mice, rats and prairie voles [2,3,4,5,6,7,8,9,10,11,12] as well as genetically obese mice and rats [including obese Zucker fatty (*fa/fa) and* Koletsky (*fa^k^/fa^k^*) rats and *ob/ob*, *db/db* and *Sim1*^+/−^ mice) [5,13,14,15,16,17,18]. This effect has been observed to some extent in male and female rodents fed chow [2,3,4,5,6,7,13,19,20,21,22,23,24,25,26] and male rodents fed purified low-fat diet [5], glucose [27], and sucrose [21,22,28], as well as male and female rodents fed a purified high-fat diet (HFD) [2,3,4,5,6,7,8,9,12,29,30]. Together, these findings have attracted widespread excitement about the potential use of OT as an anti-obesity treatment strategy and these effects have been recently translated from rodent models of diet-induced obesity (DIO) to DIO nonhuman primates [31] and overweight and obese humans [32,33,34,35].

While substantial evidence has highlighted OT’s effectiveness to reduce standard chow intake, it is not entirely clear which receptor populations throughout the forebrain and hindbrain may contribute to this effect. Acute injection studies in rats have shown that OT reduces chow intake following both forebrain (basolateral amygdala [22], ventromedial hypothalamus) [23,26], and hindbrain administration (nucleus of the solitary tract (NTS)) [24]. While additional studies have also reported that acute injections of OT into the lateral [19,20,25], third (3V) [5,15], and fourth (4V) ventricles [24,36] reduce chow intake in rats, comparisons between forebrain (3V) and hindbrain (4V) routes of administration using the same doses and paradigm were not examined. Ong and colleagues found that relatively high doses of OT appeared to be required to reduce short-term food intake following acute 4V administration [24] (3–12 μg) when compared to doses that have been effective at reducing short-term food intake following acute 4V [36] (1 μg) or lateral ventricular administration [25] (1 μg) in other studies. Therefore, we examined the dose-response effects of acute OT administration into both the 3V [which targets both forebrain and hindbrain OT receptors (OTRs)] and 4V (to target hindbrain OTRs) in order to assess the relative contribution of forebrain and hindbrain OTRs in the control of short-term food intake.

We recently found that chronic 4V OT administration is sufficient to ameliorate diet-induced obesity in CD^®^ IGS and Long-Evans rats [6,30] and C57Bl/6J mice [12], but one other important question is whether hindbrain (4V) administration may also prevent the progression of diet-induced obesity. While we recently identified that chronic 3V infusion of OT can reduce weight gain associated with diet-induced obesity in CD^®^ IGS rats, the extent to which these effects may have been mediated, in part, by activation of hindbrain OTRs is not clear. Therefore, we determined the extent to which chronic 4V OT infusion may reduce weight gain, adiposity, and energy intake associated with the progression of diet-induced obesity.

Another outstanding question is the identification of specific neuronal populations that may contribute to the effects of CNS hindbrain (4V) OT to reduce chow intake in rats. Previous studies have shown that acute lateral ventricular administration of OT increases c-Fos within the caudal NTS (cNTS) [25,37] (1 μg [25] or 10 μg [38]). In addition, Emch and colleagues found that direct application of OT (20 ng) into the NTS through an injection pipette also resulted in elevated c-Fos within the NTS of anesthetized rats [39]. However, the phenotype of these cNTS neurons activated in response to hindbrain (4V) administration of OT has not been clearly identified. Immunocytochemical and pharmacological studies suggest that glucagon-like peptide-1 (GLP-1) and proopiomelanocortin (POMC) neurons are downstream targets of OT action and contribute to the ability of OT to reduce food intake [15,25]. In addition, OT fibers that originate from the PVN are found in close anatomical proximity to NTS noradrenergic (NA) neurons [40] and we have shown that systemic OT increases Fos expression in NTS catecholamine neurons following systemic OT treatment in both mice and rats [36], but the extent to which NTS catecholamine neurons are activated in response to hindbrain (4V) OT has not been examined. Therefore, we examined the extent to which 4V OT, at a dose that suppresses chow consumption, also activates NTS catecholamine neurons.

Our findings demonstrate that acute 4V administration of OT produces a dose-dependent suppression of chow consumption. Furthermore, chronic hindbrain (4V) administration of OT reduced HFD consumption and relative body adiposity (pre vs. post OT intervention) and tended to reduce plasma leptin. Lastly, we found that 4V OT increased the number of cNTS Fos (+) neurons and induced Fos in tyrosine hydroxylase (TH; marker of catecholamine neurons) (+) neurons relative to vehicle. Collectively, these findings support the hypothesis that both acute and chronic OT administration in the hindbrain results in the suppression of food intake and that these effects may involve activation of hindbrain catecholamine neurons.

## 2. Methods

### 2.1. Animals

Adult male Sprague–Dawley (SD-SAS) rats (Charles River Laboratories International, Inc., Wilmington, MA, USA; weight range 299–435 g; ~2–3 months at time of study onset) used in the acute injection studies were maintained on a chow diet containing 13% kcal from fat [5001 (LabDiet, St. Louis, MO, USA)]. CD^®^ IGS rats (Charles River Laboratories International, Inc.; 352–528 g; ~3 months old at time of study onset) used to examine if chronic 4V infusion of OT could prevent diet-induced obesity (Study 3) were maintained on chow and then switched to a HFD containing 60% kcal from fat (Research Diets, Inc., D12492, New Brunswick, NJ, USA) at time of minipump implantations. Long Evans rats (Envigo, Indianapolis, IN, USA); 311–366 g; ~3 months old at time of study onset) were also used to determine the extent to which chronic 3V administration could also prevent diet-induced obesity and maintained on chow prior to being switched to the HFD containing 60% kcal from fat (D12492; Research Diets, Inc., New Brunswick, NJ, USA) at time of minipump implantation. The animals were housed individually in Plexiglass cages in a temperature-controlled room (21–23 °C) under a 12/12-h light–dark cycle and adapted to a 6-h fast before the start of the dark cycle (Study 1 and Study 5) or were ad libitum fed (Studies 2–4). Animals were maintained on a reversed light cycle (lights off at 1 PM). Rats had ad libitum access to water and diet unless otherwise stated. All groups were age-matched and weight-matched prior to the study onset. The research protocols were approved both by the Institutional Animal Care and Use Committee of the Veterans Affairs Puget Sound Health Care System (VAPSHCS) and the University of Washington (Animal Component of Research Protocol #s 0856 and 0928) in accordance with NIH Guidelines for the Care and Use of Animals.

### 2.2. Drug Preparation

Fresh solutions of OT acetate salt were prepared the day of each experiment (Study 1 and Study 5). OT and bombesin (Bachem Americas, Torrance, CA, USA) were solubilized in sterile water. Angiotensin II (Sigma-Aldrich, St. Louis, MO, USA) was dissolved in sterile saline and used to verify cannula placement (Study 1). Fresh solutions of OT acetate salt (Bachem Americas, Inc., Torrance, CA, USA) were solubilized in sterile water, loaded into Alzet^®^ minipumps (model 2004; DURECT Corporation, Cupertino, CA, USA), and subsequently primed in sterile 0.9% saline at 37 °C for approximately 40 h prior to minipump implantation, based on manufacturer’s recommended instructions (Study 2–4).

### 2.3. 3V and 4V Cannulations for Acute Delivery in SD-SAS Rats

Animals were implanted with a sterile cannula that was directed towards the 3V or 4V (model C315GA/SPC- 11 mm below pedestal for 3V or 9 mm below pedestal for 4V; Plastics One Inc., Roanoke, VA, USA) or 4V, as previously described [5]. Briefly, rats under isoflurane anesthesia were placed in a stereotaxic apparatus with the incisor bar positioned 3.3 mm below the interaural line. A 26-gauge cannula (Plastics One Inc.) was stereotaxically positioned into the 3V (8.1 anterior to the interaural line; 0 mm lateral to the midline, and 7.6 mm ventral to the skull surface) or 4V (3.5 posterior to bregma; 1.4 mm lateral to the midline, and 6.2 mm ventral to the skull surface) and secured to the surface of the skull with dental cement and stainless steel screws. A sterile 33-gauge dummy cap (Plastics One Inc.) was inserted into the cannula to maintain patency. Animals were injected with buprenorphine hydrochloride (0.3 mg/kg sc; Reckett and Colman Pharmaceuticals, Richmond, VA, USA) and the antibiotic enrofloxacin (5 mg/kg; Baytril, Patterson Veterinary, Devens, MA, USA) at the completion of surgery and were allowed to recover at least 7 days prior to verification of cannula placement. Cannula placement was verified in animals with 3V and 4V cannulas before the start of experiments. Cannula confirmation in rats with 3V cannulas for acute delivery was completed by measuring the drinking response following 3V injection of angiotensin II at a dose of 20 ng/μL. All rats that drank at least 5 mL of water over a 30-min period were used in the subsequent data analysis. Cannula confirmation in rats with 4V cannulas for acute delivery was completed by measuring the ability of acute 4V administration of bombesin (15 pmol/μL) to suppress short-term food intake. All rats whose food intake was suppressed by 20% over a 2-h period were used in the subsequent data analysis [41].

### 2.4. Acute 3V and 4V Injections and Measurements of Food Intake in SD-SAS Rats

OT (or saline vehicle; 1-μL injection volume) was administered immediately prior to the start of the dark cycle following 6 h of food deprivation in rats. Animals were habituated to regular handling and sham injections for at least 1 wk. prior to the commencement of studies. Third ventricular (3V) (model C315IA-SPC Acute Internal Cannula fit 11 mm C315GA; Plastics One Inc.) (1 μL) and 4V (C315IA-SPC Acute Internal Cannula fit 9 mm C315GA; Plastics One Inc.) (1 μL) injections were administered via a sterile 33-gauge injector connected by sterile polyethylene 20 tubing to a 10-μL Hamilton syringe. Injections were completed over 1 min using an injection pump (CMA 100 Syringe Pump; CMA Microdialysis AB, North Chelmsford, MA, USA); the injector was slowly removed over 10 s and replaced with a 33-gauge dummy cap (Plastics One Inc.). Animals underwent all treatment conditions (unless otherwise noted) in a randomized order, separated by at least 48 h between treatments.

### 2.5. 3V and 4V Cannulations for Chronic Delivery in CD^®^ IGS and Long–Evans Rats

Animals were implanted with a cannula within the 3V or 4V with a side port that was connected to an osmotic minipump (model 2004, DURECT Corporation, Cupertino, CA, USA). Briefly, animals under isoflurane anesthesia were placed in a stereotaxic apparatus with the incisor bar positioned 5.0 mm below the interaural line. A 26-gauge cannula (model 3280P/SPC-11 mm below pedestal for 3V or 9 mm below pedestal for 4V; Plastics One Inc.) was stereotaxically positioned into the 3V (7.3 mm anterior to the interaural line; 0 mm lateral to the midline, and 8.6 mm ventral to the skull surface; Long-Evans) or 4V (3.5 mm anterior to the interaural line; 1.4 mm lateral to the midline, and 7.2 mm ventral to the skull surface; CD^®^ IGS) and secured to the surface of the skull with dental cement and stainless steel screws. A sterile 2.4-inch piece of plastic tubing (Tygon^TM^ Microbore Tubing, 0.020” × 0.060”OD, 100 ft./roll; Cole-Parmer, Vernon Hills, IL, USA) was tunneled subcutaneously along the midline of the back and connected to the 21-gauge sidearm osmotic minipump-cannula assembly. A stainless steel 22-gauge pin plug (Instech Laboratories, Inc., Plymouth Meeting, PA, USA) was temporarily inserted at the end of the tubing during the postoperative recovery period, after which it was removed and connected to an osmotic minipump (Alzet^®^ model 2004; DURECT Corporation, Cupertino, CA, USA) containing saline or OT in a second surgical procedure. Animals were either treated with the analgesic ketoprofen (5 mg/kg; Fort Dodge Animal Health, Fort Dodge, IA, USA) or buprenorphine SR LAB [sustained release (0.65–1 mg/kg); ZooPharm, Windsor, CO, USA] and the antibiotic enrofloxacin (5 mg/kg; Baytril, Patterson Veterinary, Devens, MA, USA) at the completion of the 4V cannulations and were allowed to recover at least 10 days prior to implantation of osmotic minipumps.

### 2.6. Body Composition

Determinations of lean body mass and fat mass were made on unanesthetized, lightly restrained, chow-fed, and HFD-fed animals by quantitative magnetic resonance using an EchoMRI 4-in-1^TM^-700 instrument (Echo Medical Systems, Houston, TX, USA) at the VAPSHCS Rodent Metabolic Phenotyping Core.

## 3. Study Protocols

**Study 1: Effect of acute 3V and 4V administration of OT on food intake in male chow-fed SD-SAS rats.** SD-SAS rats were used in this study to extend previous studies that examined the effects of acute 3V administration of OT on purified low- and high-fat diet consumption [5]. On the day of the study, 6-h-fasted rats received 3V (N = 7/group) or 4V (N = 9/group) administration of OT (0.04, 0.25, and 1 μg/μL) or vehicle within 15–20 min prior to dark cycle onset. Animals received a single acute injection at 48–72-h intervals in a within-subjects design so that each animal served as its own control. A separate group of rats received vehicle or a higher dose of OT (5 μg/μL) into the 3V (N = 16/group) or 4V (N = 8/group) in identical fashion. Food was returned immediately prior to dark cycle onset and measured 0.5, 1, 2, 4, and 18 h later. Dosing was based on previously published data from our lab and others in rats [19,20,36].

**Study 2: Effect of chronic 3V OT infusions on food intake, body weight gain, and body composition in male HFD-fed Long-Evans rats**. Long-Evans rats were used in this study to extend previous studies that found chronic 3V administration of OT to reduce food intake and body weight associated with the progression of diet-induced obesity in male HFD-fed CD^®^ IGS rats [29]. 3V cannulated rats received implantations of 28-day minipumps for ventricular infusion of vehicle (saline) or OT (1.6 and 16 nmol/day). Animals were switched from chow to HFD at the time of minipump implantations and maintained on HFD for 26 days. Food intake and body weight were recorded daily in ad libitum-fed rats over 26 days. Dosing was based on recently published data from our lab and others in DIO rats [2,29].

**Study 3A: Effect of chronic 4V OT infusions on food intake, body weight gain, and body composition in male HFD-fed CD^®^ IGS rats.**

CD^®^ IGS rats were used in this study because they grow more rapidly compared with SD-SAS rats and may be a more suitable rat model to test if chronic 4V OT administration (>15 days) is effective in delaying the progression of diet-induced obesity. As mentioned earlier, we also found that chronic 3V OT administration was effective in reducing weight gain and adiposity associated with the development of diet-induced obesity in this rat model [29]. 4V cannulated rats received implantations of 28-day minipumps for ventricular infusion of vehicle (saline) or OT (16 nmol/day). Animals were switched from chow to HFD at the time of minipump implantations and maintained on HFD for 28 days. Food intake and body weight were recorded daily in ad libitum-fed rats over 28 days. Dosing was based on recently published data from our lab in DIO rats [6].

**Study 3B: Effect of chronic 4V OT infusions on plasma hormones in male HFD-fed CD^®^ IGS rats.**

Plasma hormones were measured from 4V cannulated rats that had received implantations of 28-day minipumps for ventricular infusion of vehicle (saline) or OT (16 nmol/day) (**Study 3A).**

**Study 4: Effect of chronic 4V OT infusions and post-treatment washout period on plasma hormones in established male DIO CD^®^ IGS rats.**

Data were collected from a subset of rats whose metabolic data were previously published [6] (512–885 g; ~7 months at time of study onset). In that study, 4V-cannulated rats received implantations of 28-day minipumps for ventricular infusion of vehicle (saline) or OT (16 nmol/day), and a subset of animals whose minipump was removed continued to be maintained on the HFD for an additional 27 days, as previously described, to evaluate the effect of a washout period [6]. Dosing was based on recently published data in our lab in DIO rats [6].

**Study 5: Effect of 4V administration of OT on activation of catecholamine neurons in cNTS in male chow-fed SD-SAS rats.**

### 3.1. Tissue Collection and Processing

A subset of SD-SAS rats used in Study 1 (311–374 g; ~2–3 months at time of study onset) was fasted for 6 h and received an acute 4V (5 μg/μL) injection of either OT or vehicle within 30 min prior to dark cycle onset. Animals were returned to their cages following injections and continued to have their food withheld to prevent the potential confounding effects of differences of intake on hindbrain Fos expression. Ninety minutes following injection of vehicle or OT, animals were anesthetized with a ketamine cocktail [ketamine hydrochloride (71.4 mg/kg), xylazine (3.57 mg/kg), and acepromazine (1.1 mg/kg)] and transcardially exsanguinated with saline followed by perfusion with 4% paraformaldehyde in 0.1 M PBS. Brains were removed, stored overnight in fresh fixative at 4 °C, and subsequently transferred to 0.1 M PBS containing 25% sucrose for 48 h. Brains were then frozen by submersion for 20–30 s in isopentane chilled with dry ice.

### 3.2. Immunohistochemical Staining and Quantification

Coronal cryostat sections (14 μm) of the hindbrain were mounted on slides and stored at −80 °C. Fos and TH staining was performed on anatomically matched sections throughout the NTS, as has been published previously [42]. Briefly, slides were washed at room temperature with 0.1M PBS/1% BSA followed by a blocking buffer (5% normal goat serum in 0.1M PBS) for 90 min, followed by additional buffer washes. The primary antibodies were rabbit polyclonal anti-c-Fos (Calbiochem, San Diego, CA, USA) diluted 1:5000 in 1% BSA in 0.1M PBS and mouse monoclonal anti-TH (Chemicon International Inc., Temecula, CA, USA) diluted 1:1000 in 1% BSA in 0.1M PBS. The secondary antibodies were goat anti-rabbit IgG-Cy3 (Jackson ImmunoResearch Laboratories, West Grove, PA, USA) and goat anti-mouse IgG-Alexa 488 (Life Technologies, Grand Island, NY, USA), both of which were diluted 1:200 in 1% BSA in 0.1M PBS. Control sections incubated with normal rabbit serum did not show staining. Slides were analyzed with a Nikon Eclipse 80i fluorescent microscope (Nikon Instruments, Melville, NY, USA), and all the measurements were made using a 20X objective lens. Identification of anatomic landmarks was assisted by staining cell nuclei with Hoechst 33258 (Sigma-Aldrich, St. Louis, MO, USA), which was added to the mounting medium and observed with a conventional DAPI filter set. Digital RGB images of the fluorescent preparations were acquired using a Nikon Eclipse 80i fluorescent microscope (Nikon Instruments) with Nikon NIS-Elements Software (Nikon Instruments), which included a COOLSNAPHQ2 Monochrome camera (Photometrics**^®^**, Tucson, AZ, USA), and were exported to Photoshop CS2 (Adobe, Tucson, AZ). Measurements of Fos induction in the NTS were pooled from four sections separated by 240 μm (bregma −14.16 to −13.32) according to the rat brain atlas [43]]: Level 1 (−14.16), Level 2 (−13.92), Level 3 (−13.68) and Level 4 (−13.32). In each NTS section analyzed, the number of neurons that had Fos in the nucleus was recorded bilaterally. The total number of NTS Fos (+) neurons across the four anatomically matched sections was analyzed across the treatment groups. The number of Fos (+) cells in the NTS was derived from the cumulative number of Fos (+) cells between the treatments across four sections for the NTS.

## 4. Adipose Tissue Processing for Adipocyte Size and UCP-1 Analysis

Inguinal white adipose tissue (IWAT) and epididymal white adipose tissue (EWAT) depots were collected at the end of the infusion period in HFD-fed CD^®^ IGS rats from Study 3. Rats were fasted for 3 h prior to being euthanized with intraperitoneal injections of a ketamine cocktail [ketamine hydrochloride (214.3 mg/kg), xylazine (10.71 mg/kg), and acepromazine (3.3 mg/kg) in an injection volume up to 1 mL/rat] and transcardially exsanguinated with PBS followed by perfusion with 4% paraformaldehyde in 0.1 M PBS. Adipose tissue (IWAT and EWAT) was dissected and placed in 4% paraformaldehyde-PBS for 24 h and then placed in 70% ethanol (EtOH) prior to paraffin embedding. Sections (5 μm) sampled were obtained using a rotary microtome, slide-mounted using a floatation water bath (37 °C), and baked for 30 min at 60 °C to give approximately 15–16 slides/fat depot with two sections/slide. 

## 5. Adipocyte Size Analysis and UCP-1 Staining

Adipocyte size analysis was performed on deparaffinized and digitized IWAT and EWAT sections. The average cell area from two randomized photomicrographs was determined using the built-in particle counting method of ImageJ software (National Institutes of Health, Bethesda, MD, USA). Fixed (4% PFA), paraffin-embedded adipose tissue was sectioned and stained with a primary rabbit anti-UCP-1 antibody (1:100; Abcam, Cambridge, MA, USA (#ab10983/RRID: AB_2241462)], as has been previously described in lean C57BL/6J mice [44] and both lean and DIO C57BL/6 mice after having been screened in both IBAT and IWAT of Ucp1^+/−^ and Ucp1^−/−^ mice [45]. Immunostaining specificity controls included omission of the primary antibody and replacement of the primary antibody with normal rabbit serum at the same dilution as the respective primary antibody. Area quantification for UCP1 staining was performed on digital images of immunostained tissue sections using image analysis software (Image Pro Plus software, Media Cybernetics, Rockville, MD, USA). Slides were visualized using bright field on an Olympus BX51 microscope (Olympus Corporation of the Americas; Center Valley, PA, USA) and photographed using a Canon EOS 5D SR DSLR (Canon U.S.A., Inc., Melville, NY, USA) camera at 100X magnification. Values for each tissue within a treatment were averaged to obtain the mean of the treatment group.

## 6. Blood Collection

Blood was collected in 3-h fasted rats (Studies 3–4) at the end of the light cycle within a 2-h window (10:00 a.m.–12:00 p.m.), as previously described [6,29]. Treatment groups were counterbalanced at the time of euthanasia to avoid bias. Blood samples (3 mL) were collected immediately prior to transcardial perfusion by cardiac puncture in chilled K2 EDTA Microtainer Tubes (Becton-Dickinson, Franklin Lakes, NJ, USA). Whole blood was centrifuged at 6000 rpm for 1.5 min at 4 °C; plasma was removed, aliquoted, and stored at −80 °C for subsequent analysis.

## 7. Plasma Hormone Measurements

Plasma leptin and insulin were measured using electrochemiluminescence detection Meso Scale Discovery (MSD^®^, Rockville, MD, USA) using established procedures [6,12,29,30,46]. Intra-assay coefficient of variation (CV) for leptin was 2.7% and 3.2% for insulin. The range of detectability for the leptin assay was 0.137–100 ng/mL and 0.069–50 ng/mL for insulin. Plasma fibroblast growth factor-21 (FGF-21) (R&D Systems, Minneapolis, MN, USA) and irisin (AdipoGen, San Diego, CA, USA) levels were determined by ELISA. The intra-assay CV for FGF-21 and irisin were 4.5% and 8.4%, respectively; the ranges of detectability were 31.3–2000 pg/mL (FGF-21) and 0.078–5 μg/mL (irisin). Plasma adiponectin was also measured using electrochemiluminescence detection Meso Scale Discovery (MSD^®^, Rockville, MD, USA) using established procedures [6,46]. Intra-assay CV for adiponectin was 1.1%. The range of detectability for the adiponectin assay was 2.8–178 ng/mL. The data were normalized to historical values using a pooled plasma quality control sample that was assayed in each plate.

## 8. Blood Glucose and Lipid Measurements

Whole blood was collected in 3-h fasted rats for glucose measurements by tail vein nick and measured with a glucometer using the AlphaTRAK 2 blood glucose monitoring system (Abbott Laboratories, Abbott Park, IL, USA) [47]. Total plasma cholesterol (TC) [Fisher Diagnostics (Middletown, VA, USA)] and free fatty acids (FFAs) [Wako Chemicals USA, Inc., Richmond, VA, USA)] were measured using an enzymatic-based kits. Intra-assay CVs for TC and FFAs were 2.8% and 2.5%, respectively. These assay procedures have been validated for rodents [48].

## 9. Statistical Analyses

All results are expressed as means ± SE. Comparisons between multiple groups involving between-subjects designs were made using one-way or two-way ANOVA followed by a post hoc Fisher’s least significant difference test. Comparisons involving within-subjects designs were made using a one-way repeated-measures ANOVA followed by a post hoc Fisher’s least significant difference test. Analyses were performed using the statistical program SYSTAT (Systat Software, Point Richmond, CA, USA). Differences were considered significant at *P* < 0.05, 2-tailed.

## 10. Results

**Study 1: Effect of acute 3V and 4V administration of OT on food intake in male chow-fed SD-SAS rats.**

**3V:** OT (5 μg) significantly reduced food intake at 0.5 h (F(1,15) = 63.171, *P* < 0.05), 1 h (F(1,15) = 38.479, *P* < 0.050), 3 h (F(1,15) = 10.939, *P* < 0.05), and 4 h post-injection (F(1,15) = 8.015, *P* < 0.05). 3V OT (5 μg) suppressed 0.5-, 1-, 3-, and 4-h food intake by 71.7 ± 6.0, 49.3 ± 6.9, 15.7 ± 6.3, and 12.5 ± 5.4%, respectively (*P* < 0.05). 3V OT (5 μg) did not significantly reduce food intake at 2 h post-injection (*P* = 0.155).

**Figure 1:**
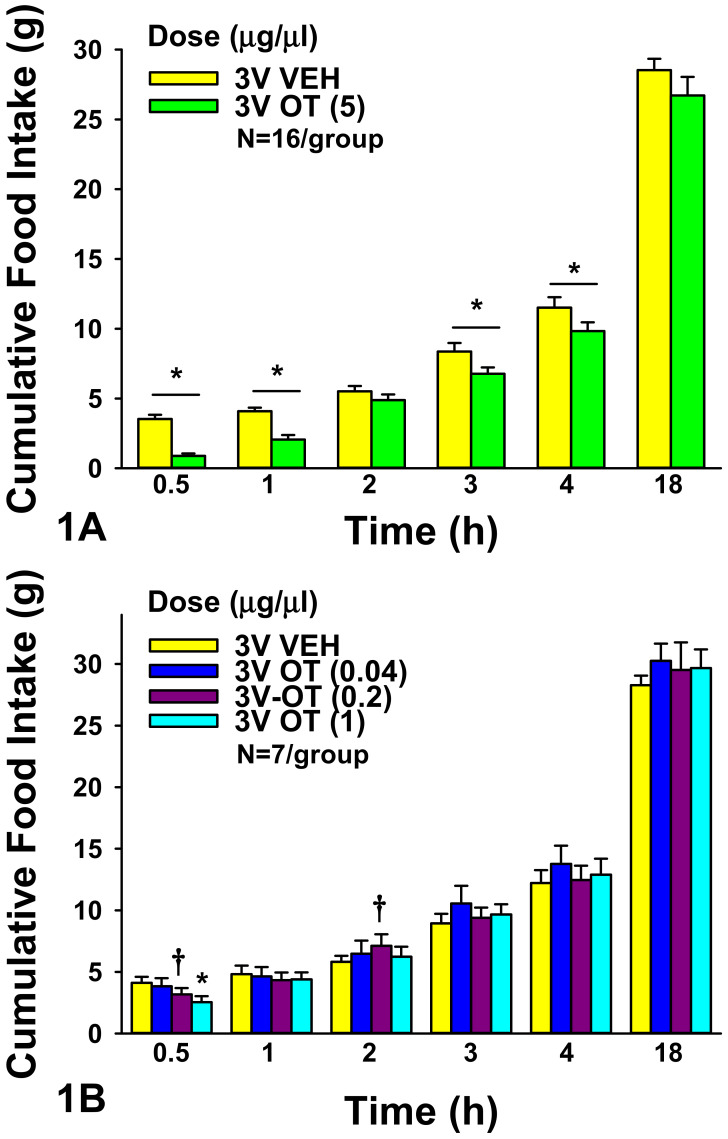
(**A**,**B**), **Effect of acute 3V administration of OT on food intake in male chow-fed SD-SAS rats.** (**A**), Food intake was measured in rats that received acute 3V injections of OT (5 μg/μL) or vehicle (N = 16/group). (**B**), Food intake was measured in rats that received acute 3V injections of OT (0.04, 0.2, 1 μg/μL; N = 7/group). Data are expressed as mean ± SEM. * *P* < 0.05 or † 0.05 < *P* < 0.1 OT vs. vehicle.

We also examined the effect of lower doses of OT to suppress food intake in a separate group of rats. There was an overall significant dose effect of 3V OT to reduce food intake 0.5 h (F(3,18) = 3.397, *P* < 0.05). 3V OT (1 μg) suppressed 0.5-h food intake by 38.3 ± 10.9% (*P* < 0.05). 3V OT (0.2 μg) tended to reduce 0.5-h food intake by 23.7 ± 12.0% (0.05 < *P* < 0.1; Figure 1A,B) but also tended to stimulate 2-h intake by 25.7 ± 12.0% (0.05 < *P* < 0.1). The tendency for the low dose of OT to stimulate food intake was unexpected and might have been a compensatory effect to offset the effects of the low dose to reduce 0.5-h food intake.

**4V:** OT (5 μg) significantly reduced food intake at 0.5 h (F(1,7) = 8.933, *P* < 0.05), 1 h (F(1,7) = 12.522, *P* < 0.05), and 4 h post-injection (F(1,7) = 7.969, *P* < 0.05) by 60 ± 12.9, 43.5 ± 9.5, and 18.2 ± 7.3%, respectively (*P* < 0.05). 

**Figure 2:**
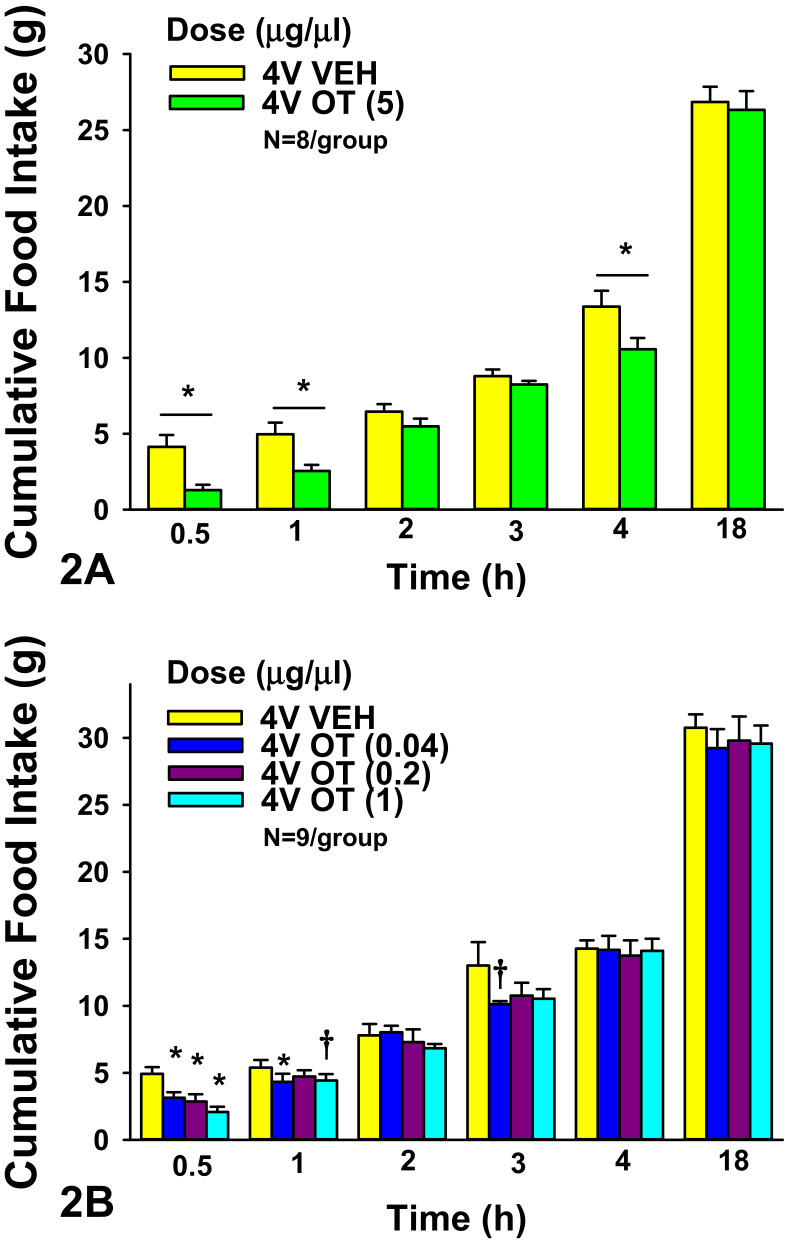
(**A**,**B**), **Effect of acute 4V administration of OT on food intake in male chow-fed SD-SAS rats.** (**A**), Food intake was measured in rats that received acute 4V injections of OT (5 μg/μL) or vehicle (N = 8/group). (**B**), Food intake was measured in rats that received acute 4V injections of OT (0.04, 0.2, 1 μg/μL; N = 9/group). Data are expressed as mean ± SEM. * *P* < 0.05 or † 0.05 < *P* < 0.1 OT vs. vehicle.

We also examined whether lower doses of 4V OT would also be effective at suppressing short-term food intake in a separate group of rats. There was an overall significant dose effect of 4V OT to reduce food intake at 0.5 h (F(3,21) = 9.105, *P* < 0.05). 4V administration of OT inhibited 0.5-h food intake by 30.9 ± 12.9, 42.1 ± 9.4, and 56.4 ± 9.0% at 0.04, 0.2, and 1 μg relative to vehicle-treated animals (*P* < 0.05; Figure 2A,B). 4V OT (0.04 μg) was also effective at reducing food intake at 1 h post-injection by 16.8 ± 12.4% (*P* < 0.05) while the slightly higher dose (1 μg) tended to reduce 1-h food intake by 15.0 ± 9.9% (0.05 < *P* < 0.01). 4V OT (0.04 μg) also tended to reduce food intake by 15.8 ± 7.4% at 3 h post-injection (0.05 < *P* < 0.1).

Comparisons between 3V and 4V OT administration sites

There was no difference in the percent of food intake suppression following acute 3V or 4V administration of the high dose (5 μg) at 0.5 (F(1,22) = 0.890, *P* = NS), 1-(F(1,22) = 0.239, *P* = NS) and 4 h (F(1,22) = 0.378, *P* = NS) post-injection. Likewise, there was also no difference in the percent of food intake suppression following acute 3V or 4V administration of OT at 0.04 (F(1,13) = 1.256, *P* = NS), 0.2 (F(1,13) = 1.493, *P* = NS), or 1 μg (F(1,13) = 1.672, *P* = NS) at 0.5 h post-injection. 

**Figure 3:**
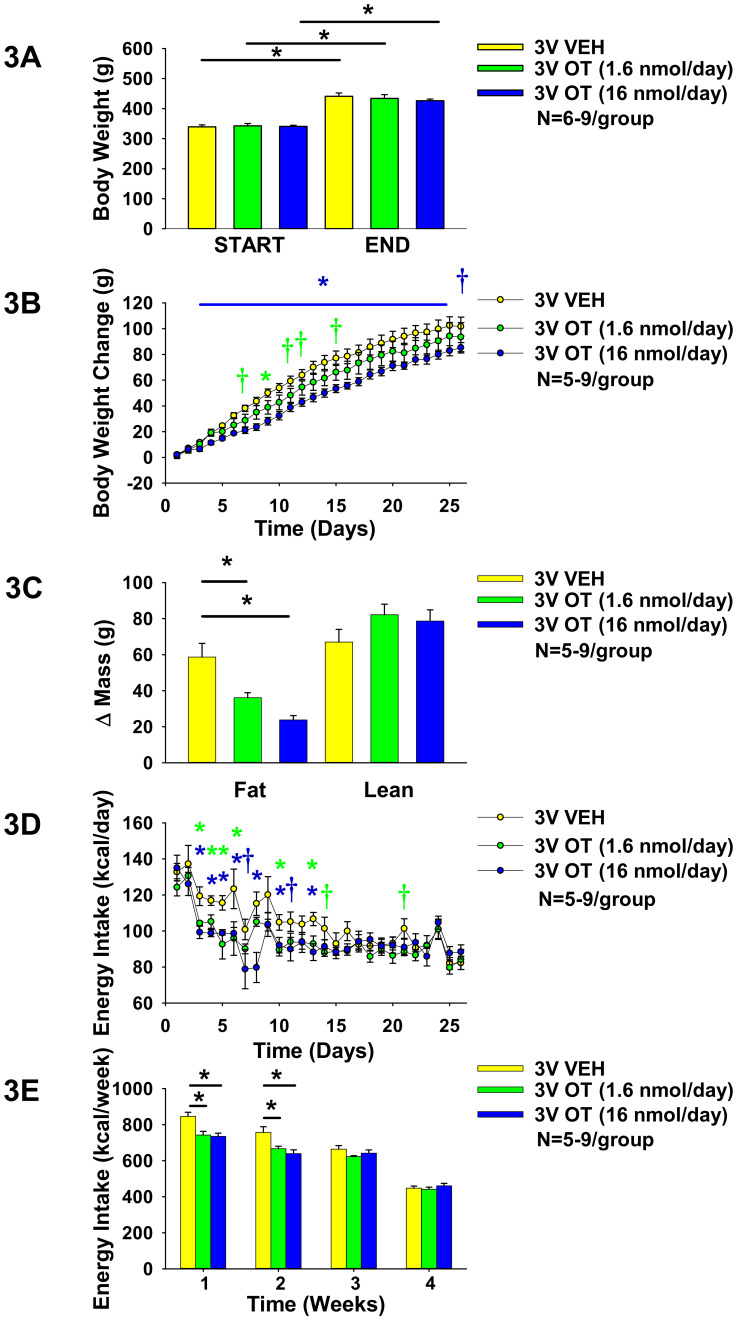
(**A**–**E**). Effects of chronic 3V OT infusions (1.6 or 16 nmol/day) on food intake, body weight gain, and body composition in male HFD-fed Long–Evans rats. Ad libitum-fed rats were placed on HFD (60% kcal from fat (Research Diets, Inc., D12492); N = 5–9/group) at onset of continuous 3V infusions of vehicle or OT (16 nmol/day). (**A**), Body weight before and after chronic 3V treatment in animals maintained on the HFD. (**B**), Cumulative change in body weight throughout course of chronic 3V treatment in animals maintained on the HFD. (**C**), Change in fat mass and lean mass before and after chronic 3V treatment in animals maintained on HFD. (**D**), Change in daily energy intake (kcal/day) in animals maintained on HFD. (**E**), Cumulative change in weekly energy intake (kcal/week) in animals maintained on HFD. Note week 4 data represent energy intake data over 5 days. Data are expressed as mean ± SEM. * *P* < 0.05 OT vs. vehicle; † 0.05 < *P* < 0.1 OT (1.6 nmol/day) vs vehicle; * *P* < 0.05 OT (1.6 nmol/day) vs. vehicle; † 0.05 < *P* < 0.1 OT (16 nmol/day) vs vehicle; * *P* < 0.05 OT (16 nmol/day) vs. vehicle.

**Study 2: Effect of chronic 3V OT infusions on food intake, body weight gain, and body composition in male HFD-fed Long-Evans rats.** Our previous work demonstrated that chronic 3V OT administration is sufficient to prevent and reverse DIO in CD^®^ IGS rats [29]. To determine if this effect is consistent across other DIO rat models, we studied Long-Evans rats, which exhibit increased susceptibility to DIO. To probe the role of forebrain OTRs in preventing weight gain associated with diet-induced obesity in Long–Evans rats, we determined the dose-response effects of chronic 3V OT (to target forebrain OTRs) on body weight gain in HFD-fed Long–Evans rats. We found that 3V infusions of OT (1.6 and 16 nmol/day) produced dose-dependent reductions of weight gain and adiposity gain in HFD-fed rats in a diet-induced obesity prevention paradigm (N = 5–9/group). Repeated measures ANOVA (3V infusion days 3, 26) indicated there was a significant main effect of 3V OT to reduce weight gain ((F(1,15) = 5.439, *P* < 0.05). As before in HFD-fed CD^®^ IGS rats [29], OT did not elicit weight loss in this paradigm (Figure 3A) but was effective at reducing weight gain at the high dose (16 nmol/day) between infusion days 3–25 (*P* < 0.05) and on infusion day 26 (0.05 < *P* < 0.1) (Figure 3B). This effect to limit weight gain was associated with reductions in adiposity, with no effect on lean mass (pre vs. post intervention; *P* < 0.05; Figure 3C), and largely recapitulated what we found following chronic 3V infusions in HFD-fed CD^®^ IGS rats [29]. The effects at the high dose (16 nmol/day) were accompanied by transient reductions of energy intake across weeks 1–2 (*P* < 0.05) and specifically on infusion days 3–6 (*P* < 0.05), 7 (0.05 < *P* < 0.1), 8 (*P* < 0.05), 10 (*P* < 0.05), 11 (0.05 < *P* < 0.01), and 13 (*P* < 0.05) (Figure 3D–E). Infusions at the lower dose (1.6 nmol/day) also produced transient reductions in energy intake over weeks 1–2 (*P* < 0.05). 3V OT reduced energy intake on infusion days 3–6, 10, and 13 (*P* < 0.05) and tended to suppress energy intake on infusion days 14 and 21 (0.05 < *P* < 0.1). Note that food intake data were not collected from a subset of rats on infusion days 12–13 (N = 1 OT (16 nmol/day)) and days 25–26 (N = 1 OT (1.6 nmol/day)), which may have impacted the food intake data on these particular days. 3V OT at either 1.6 nmol/day (125.6 ± 5.9 mg/dL) or 16 nmol/day (147.7 ± 5.4 mg/dL) also did not result in any significant changes in blood glucose relative to vehicle-infused Long-Evans rats (149.4 ± 8.5 mg/dL; *P* = NS).

**Figure 4:**
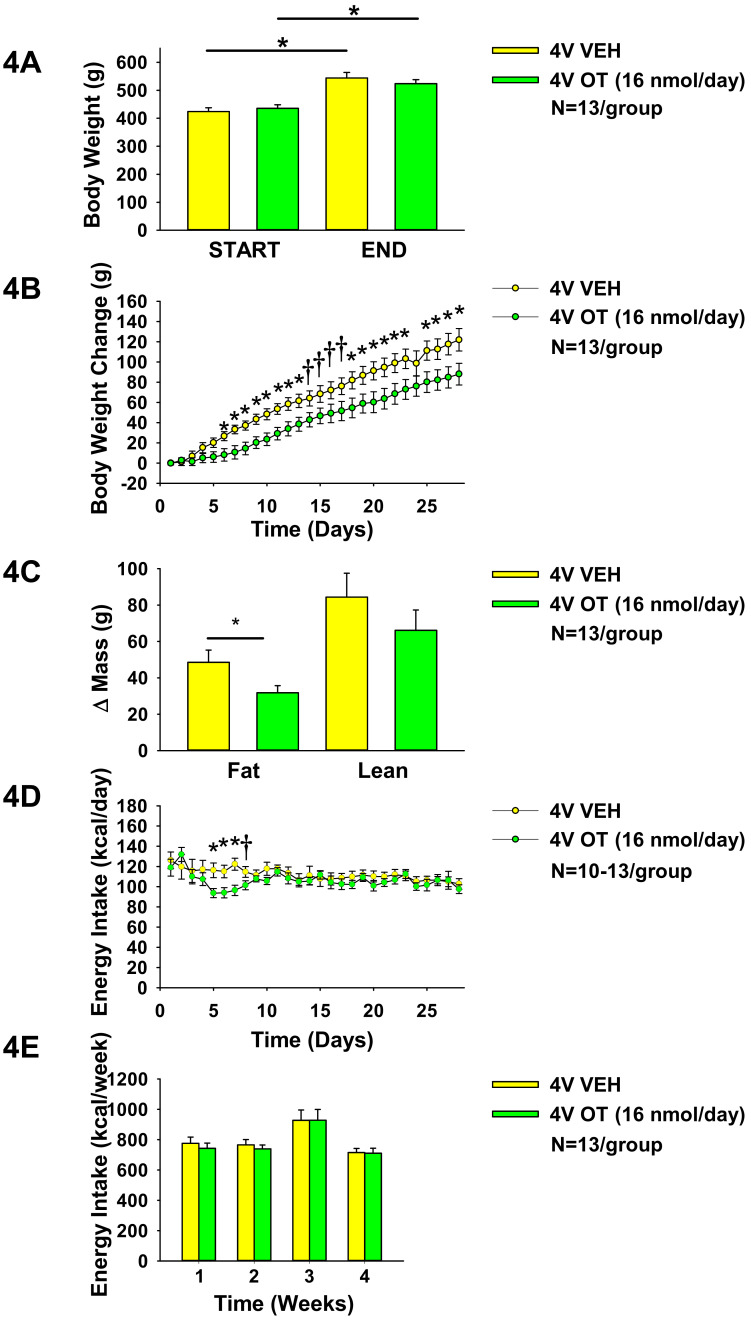
(**A**–**E**). Effects of chronic 4V OT infusions (16 nmol/day) on food intake, body weight gain, and body composition in male HFD-fed CD^®^ IGS rats. Ad libitum-fed rats were placed on HFD [60% kcal from fat (Research Diets, Inc., D12492); N = 13/group] at onset of continuous 4V infusions of vehicle or OT (16 nmol/day). (**A**), Body weight before and after chronic 4V treatment in animals maintained on the HFD. (**B**), Cumulative change in body weight throughout course of chronic 4V treatment in animals maintained on the HFD. (**C**), Change in fat mass and lean mass before and after chronic 4V treatment in animals maintained on HFD. (**D**), Change in daily energy intake (kcal/day) in animals maintained on HFD. (**E**), Cumulative change in weekly energy intake (kcal/week) in animals maintained on HFD. Data are expressed as mean ± SEM. * *P* < 0.05 or † 0.05 < *P* < 0.1 OT vs. vehicle.

**Study 3A: Effects of chronic 4V OT infusions on food intake, body weight gain, and body composition in male HFD-fed CD^®^ IGS rats.** Our previous work demonstrated that chronic 4V OT administration is sufficient to reverse DIO in both CD^®^ IGS and Long–Evans rats [6,29]. To probe the role of hindbrain OTRs and their role in preventing weight gain associated with DIO in CD^®^ IGS rats, we determined the effects of chronic 4V OT (to target hindbrain OTRs) on body weight gain in HFD-fed CD^®^ IGS rats. We found that 4V infusions of OT (16 nmol/day) reduced weight gain and adiposity gain in HFD-fed rats in a diet-induced obesity prevention paradigm (N = 13/group). Repeated-measures ANOVA (4V infusion days 5,28) indicated there was a significant main effect of chronic 4V OT treatment to reduce weight gain ((F(1,24) = 5.266, *P* < 0.05). Similar to what we observed in HFD-fed Long-Evans rats (Study 2) and in HFD-fed CD^®^ IGS rats that received 3V infusions [29], OT did not elicit weight loss in this paradigm (Figure 4A), but was effective at reducing weight gain (pre vs. post intervention; 16 nmol/day). This effect to limit weight gain was driven by reductions in adiposity (pre vs. post intervention; *P* < 0.05; Figure 4C) and had no effect on lean mass. Reductions in weight gain were first evident on infusion days 5 (0.05 < *P* < 0.1), 6–13 (*P* < 0.05), 14–17 (0.05 < *P* < 0.1), 18–23, and 25–28 (*P* < 0.05; Figure 4B) and these effects were accompanied by transient reductions of energy intake on infusion days 5–7 (*P* < 0.05) and 8 (0.05 < *P* < 0.1; Figure 4D), although this did not impact overall weekly energy intake (Figure 4E). Note that food intake data were not collected from a subset of vehicle-(day 10 (N = 3 vehicle)) and OT-infused rats (days 4, 9, 10, and 25 (N = 1–2 OT)), which may have impacted the food intake data on these particular days. In one case, an animal in the OT treatment group ground its HFD and the data on those days had to be discarded.

### 10.1. Adipocyte Size

**Figure 5:**
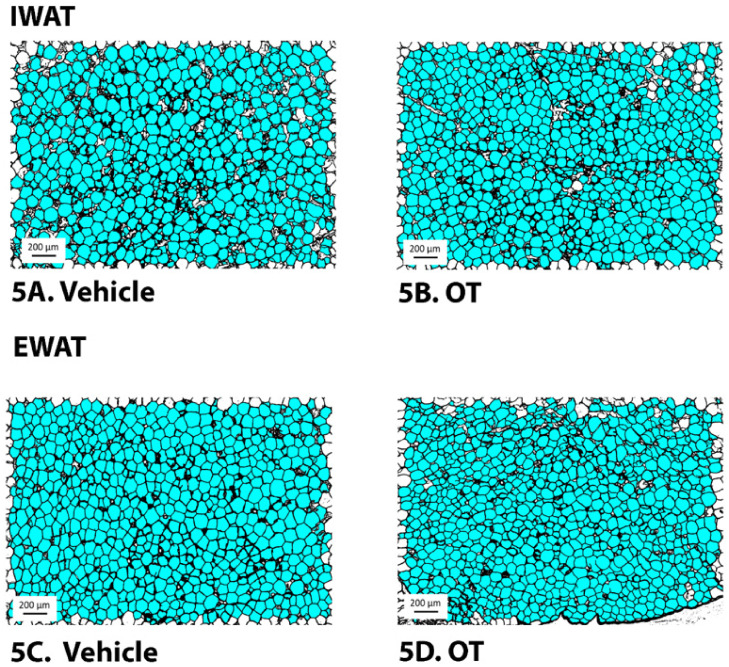
(**A**–**D**): Effect of chronic 4V OT infusions (16 nmol/day) on adipocyte size in IWAT and EWAT in male HFD-fed CD^®^ IGS rats. Adipocyte size was analyzed using ImageJ. Images were taken from fixed (4% PFA) paraffin-embedded sections (5 μm) containing IWAT (**A**,**B**) or EWAT (**C**,**D**) in HFD-fed rats treated with 4V OT (16 nmol/day) or 4V vehicle. (**A**), Veh (IWAT). (**B**), OT (IWAT). (**C**), Veh (EWAT). (**D**), OT (EWAT); (**A**–**D**) all visualized at 100× magnification.

**Figure 6:**
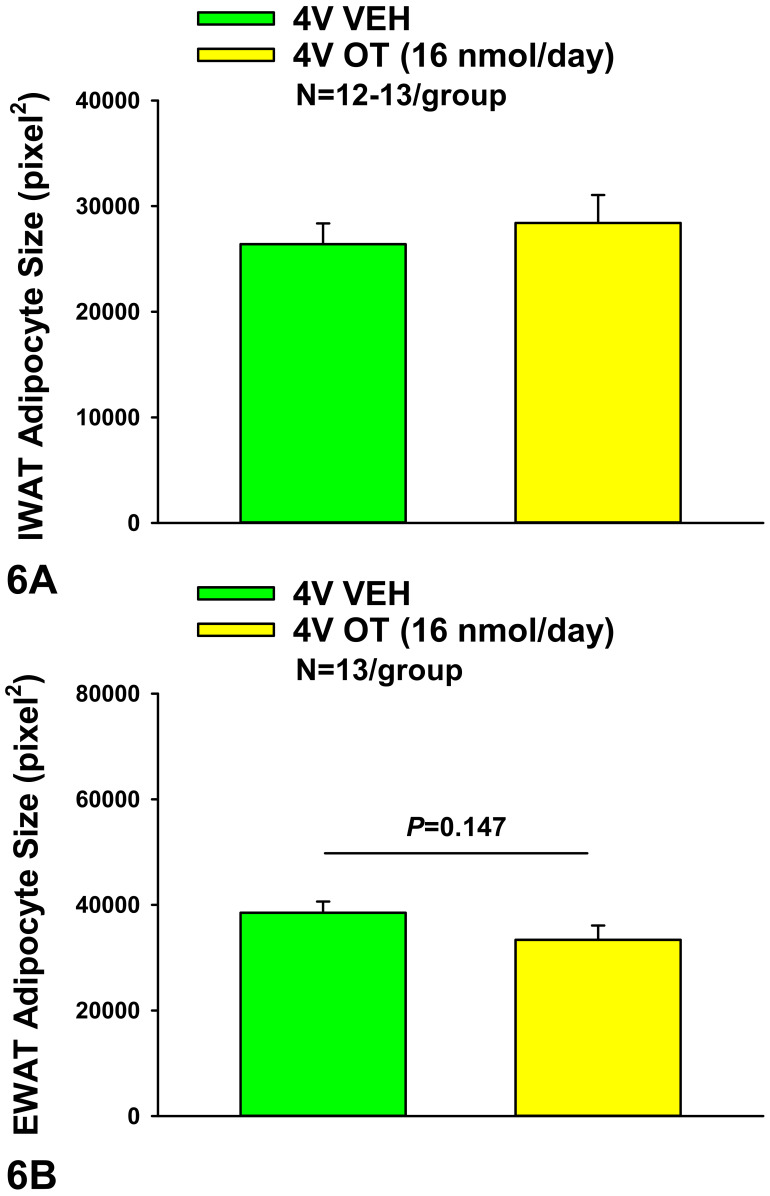
(**A**,**B**): Effect of chronic 4V OT infusions (16 nmol/day) on adipocyte size in IWAT and EWAT in male HFD-fed CD^®^ IGS rats. Adipocyte size (pixel^2^) was measured in (**A**), IWAT and (**B**), EWAT from rats that received chronic 4V infusion of vehicle or OT (16 nmol/day) (N = 13/group). Data are expressed as mean ± SEM.

Consistent with our previously published findings in DIO Long-Evans rats [30], chronic 4V OT failed to significantly impact IWAT adipocyte size in HFD-fed CD^®^ IGS rats (*P* = NS) (Figure 5A,B and Figure 6A). OT tended to reduce EWAT adipocyte size in HFD-fed CD^®^ IGS rats, although this did not reach significance (*P* = 0.148; Figure 5C,D and Figure 6B).

### 10.2. UCP-1 Expression 

**Figure 7:**
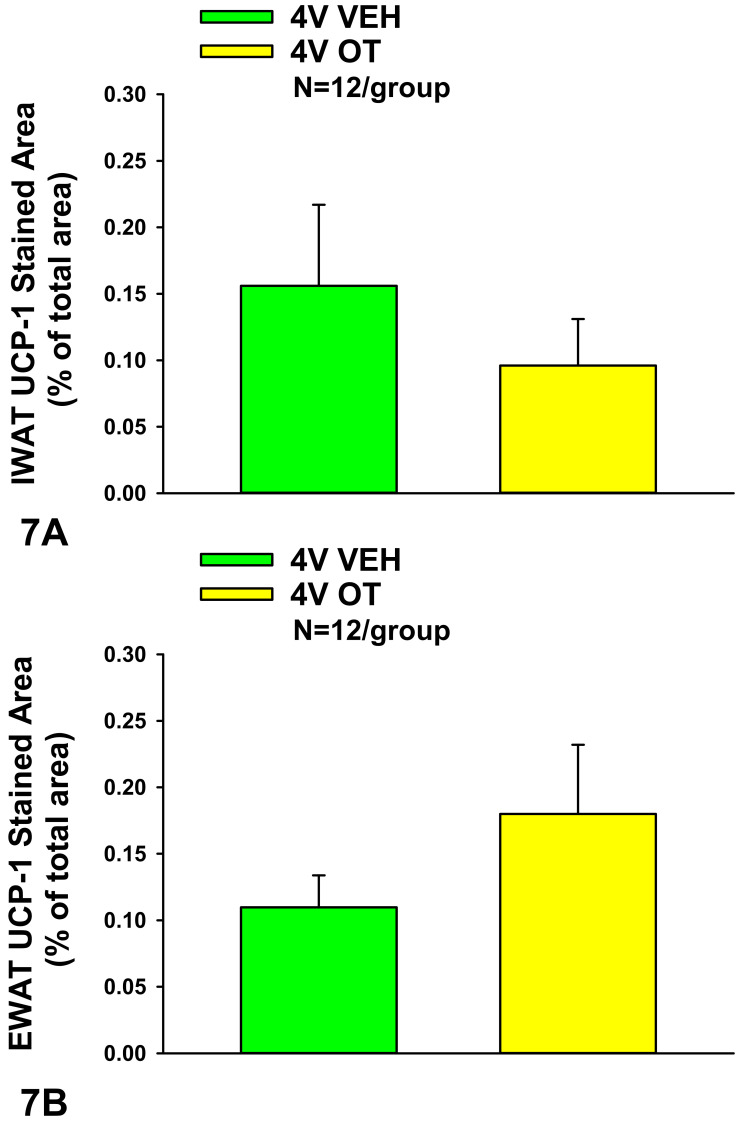
(**A**,**B**): Effect of chronic 4V OT infusions (16 nmol/day) on UCP-1 content in IWAT and EWAT in male HFD-fed CD^®^ IGS rats. (**A**), UCP-1 staining was quantified in IWAT from rats that received chronic 4V infusion of OT (16 nmol/day) or vehicle (N = 12/group). (**B**), UCP-1 staining was quantified in EWAT from rats that received chronic 4V infusion of OT (16 nmol/day) or vehicle (N = 12/group). Data are expressed as mean ± SEM.

Consistent with our previously published findings in DIO Long-Evans rats [30], chronic 4V OT failed to significantly impact increase UCP-1 in IWAT or EWAT relative to VEH (*P* = NS) in HFD-fed CD^®^ IGS rats (*P* = NS) (Figure 7A,B).

**Study 3B: Effect of chronic 4V OT infusions on plasma hormones in male HFD-fed CD^®^ IGS rats.**

To characterize the endocrine and metabolic effects of 4V OT (16 nmol/day) in HFD-fed CD^®^ IGS rats (Study 3A), we measured blood glucose levels and plasma concentrations of leptin, insulin, adiponectin, FGF-21, irisin, TC, and FFAs (Table 1). 4V OT did not result in any significant changes in plasma leptin (*P* = 0.110), insulin, adiponectin, FGF-21, irisin, FFA, or TC (*P* = NS).

**Study 4: Effect of chronic 4V OT infusions and post-treatment washout period on plasma hormones in established male DIO CD^®^ IGS rats.**

To determine whether OT elicits more beneficial metabolic effects on plasma markers in CD^®^ IGS rats with established DIO, we measured blood glucose levels and plasma concentrations of leptin, insulin, adiponectin, FGF-21, irisin, TC, and FFAs in DIO CD^®^ IGS rats from a previous publication in which chronic 4V OT was found to elicit weight loss [6]. In addition, we measured the extent to which changes in plasma hormones could be reversed following a washout period in animals from a previous publication [6]. Consistent with the ability of chronic 4V OT to reduce body weight and adiposity in DIO CD^®^ IGS rats [6], OT reduced plasma leptin (*P* < 0.05; Table 2A). However, 4V OT did not produce significant changes in plasma insulin (*P* = 0.061), adiponectin, FGF-21, irisin, FFA, or TC (*P* = NS; Table 2A). There was no change in any of the plasma markers between rats that had been treated with OT or vehicle at the conclusion of the washout period (*P* = NS; Table 2B). 

**Study 5. Effect of acute 4V administration of OT to activate catecholamine neurons in the cNTS in male chow-fed SD-SAS rats**.

**Figure 8:**
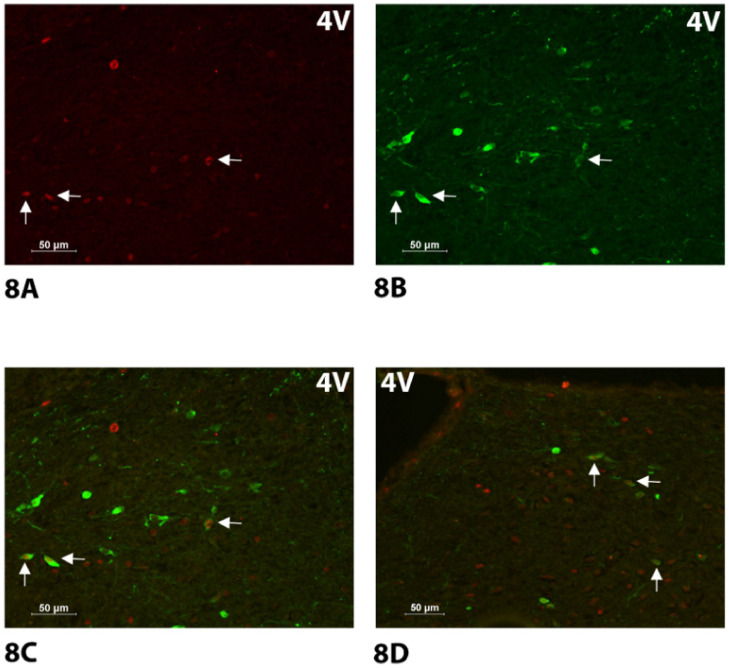
(**A**–**D**). Effect of acute 4V OT administration on Fos induction in cNTS catecholamine neurons using immunocytochemistry in male chow-fed SD-SAS rats. Neuronal activation is revealed by concentration of immunoreactive Fos in cell nuclei [Fos (+)] in the cNTS. Fos- and TH-immunostaining were done by Cy3 fluorescence and Alexa 488 fluorescence, respectively. Images were taken from one side of the cNTS. (**A**), Acute 4V administration of OT increased Fos (+) neurons in cNTS (see arrows). (**B**), TH staining in cNTS from an animal treated with 4V OT (see arrows). (**C**), Colocalization of TH (+) and Fos (+) neurons in cNTS from an animal treated with 4V OT (see arrows). (**D**), Colocalization of TH (+) neurons and Fos (+) neurons in the cNTS from the opposite side (see arrows) ((Bregma −13.44; [43]) same animal as in (**A**–**C**)); (**A**–**D**) all visualized at 20× magnification.

**Figure 9:**
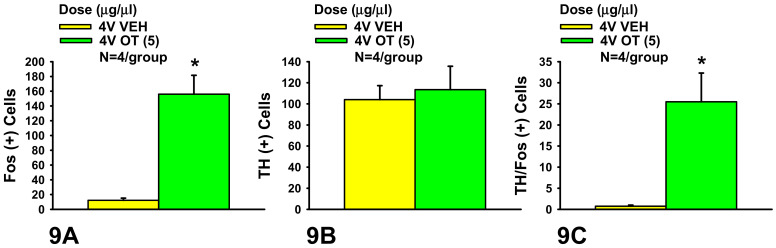
(**A**–**C**). Effect of acute 4V OT administration on Fos induction in cNTS catecholamine neurons in male chow-fed SD-SAS rats. A, Acute 4V administration of OT increased the number of Fos (+) cells in the cNTS but had no impact on B, the number of TH (+) cells. C, Acute 4V OT treatment increased the number of cNTS neurons that expressed both TH and Fos (N = 4/group). Data are expressed as mean ± SEM. * *P* < 0.05 OT vs. vehicle.

As expected, there was no effect of acute 4V OT treatment on the change in the number of TH neurons (F(1,6) = 0.135, *P* = NS). Consistent with published findings following lateral ventricular [25,37] or direct application of OT into the NTS [39] in conscious and anesthetized rats, respectively, there was a significant effect of acute 4V OT on the number of Fos (+) neurons in the cNTS (F(1,6) = 31.275, *P* < 0.05). Specifically, acute 4V administration of OT (5 μg) induced a robust 13-fold increase in the numbers of Fos+ neurons (156 ± 25) relative to vehicle (12 ± 3) across the cNTS (Figure 8A–D and Figure 9A). In addition, there was a significant effect of acute 4V OT to induce Fos in TH (+) neurons (F(1,6) = 13.227, *P* < 0.05). Administration of OT into the 4V also induced Fos in one quarter of TH+ neurons (25.5 ± 7%) relative to saline administration (0.8 ± 0.3%) (*P* < 0.05; Figure 8A–D and Figure 9B,C). Consistent with what we previously reported following systemic administration of OT in mice (10%) [36] and rats (15%) [36], the proportion of the Fos (+) NTS cells that also showed TH+ immunostaining following acute 4V OT administration was relatively small (16 ± 4%) but tended to be greater than that produced by acute 4V injection of saline (5 ± 2%) (F(1,6) = 5.812, *P* = 0.053) (Figure 9C).

Specifically, OT elicited c-Fos within the NTS at level 1 (F(1,6) = 159.402, *P* < 0.05), level 2 (F(1,6) = 11.818, *P* < 0.05], level 3 (F(1,6) = 14.384, *P* < 0.05), and level 4 (F(1,6) = 12.796, *P* < 0.05). OT also elevated c-Fos within TH neurons within the NTS at level 1 (F(1,6) = 5.825, *P* = 0.052), level 2 (F(1,6) = 14.641, *P*,0.05), and level 3 (F(1,6) = 14.832, *P*,0.05) but it did not produce a significant elevation of c-Fos within TH neurons at NTS level 4 (F(1,6) = 3.251, *P* = 0.121).

Two-way ANOVA revealed a significant overall effect of rostro-caudal level (levels 1–4) on expression of TH within the NTS (F(3,24) = 6.780, *P* < 0.05) but did not reveal any overall effect of dose (OT) on expression of TH within the NTS (F(1,24) = 0.382, *P* = NS) or any significant interactive effect between level and dose (OT) within the NTS (F(3,24) = 0.317, *P* = NS). In addition, two-way ANOVA revealed a significant overall effect of dose (OT) (F(1,24) = 64.680, *P* < 0.05) on OT-elicited c-Fos within the NTS but failed to reveal a significant effect of rostro-caudal level (levels 1–4) (F(3,24) = 0.103, *P* = NS) or interactive effect between level and dose on c-Fos (F(3,24) = 0.108, *P* = NS) within the levels of analysis within the NTS. Two-way ANOVA also revealed a significant overall effect of dose (OT) (F(1,24) = 30.281, *P* < 0.05) on Fos induction within NTS TH neurons but failed to reveal a significant effect of rostro-caudal level (F(3,24) = 0.440, *P* = NS) or interactive effect between level and dose on c-Fos in TH neurons (F(3,24) = 0.596, *P* = NS). Together, these findings suggest that there was a notable change in TH expression throughout the levels of analysis within the NTS (in the absence of treatment) and a significant effect of OT to increase c-Fos within NTS TH neurons but there was no preferential effect of OT to enhance c-Fos within TH neurons that varied according to level. 

## 11. Discussion

The goals of the current study were to examine (1) the dose-response effects of acute administration of OT into the 3V (forebrain) and 4V (hindbrain) to assess sensitivity to OT in forebrain and hindbrain sites, (2) the extent to which chronic 4V administration of OT reduces weight gain associated with diet-induced obesity, and (3) whether NTS catecholamine neurons are downstream targets of 4V OT. Previous studies by Ong and colleagues demonstrated that relatively high doses of OT appeared to be required to reduce short-term food intake following acute 4V administration [24] (3–12 μg) relative to doses that have been effective at reducing short-term food intake following acute 4V [36] (1 μg) or lateral ventricular administration [25] (1 μg) in other studies. We therefore examined the dose-response effects of 3V and 4V OT administration (0.04, 0.2, 1, or 5 μg) in lean, chow-fed rats using the same paradigm. 3V and 4V OT (5 μg) suppressed 0.5-h food intake by 71.7 ± 6.0% and 60 ± 12.9%, respectively. 4V OT (0.04, 0.2, 1 μg) reduced food intake by 30.9 ± 12.9, 42.1 ± 9.4, and 56.4 ± 9.0%, respectively, whereas 3V administration of OT (1 μg) reduced food intake by 38.3 ± 10.9%. When given chronically into the 3V and 4V of HFD-fed rats, OT was able to reduce weight gain and adiposity. These effects following chronic 4V treatment in CD^®^ IGS were accompanied by transient reductions of energy intake on infusion days 5–7 (*P* < 0.05) and 8 (0.05 < *P* < 0.1), while chronic 3V treatment in Long–Evans rats was accompanied by more persistent reductions of energy intake over the first 2 weeks of the infusion period. Lastly, we found that acute hindbrain (4V) administration of OT increased the number of cNTS Fos (+) neurons (156 ± 25) relative to vehicle (12 ± 3). 4V OT also induced Fos in TH (+) neurons (25 ± 7%) relative to vehicle (0.8 ± 0.3%). Collectively, these findings support the hypothesis that OT within the hindbrain is effective at reducing food intake, weight gain, and adiposity and that NTS catecholamine neurons in addition to non-catecholaminergic neurons are downstream targets of CNS OT.

To our knowledge, this is the first time that the effects of OT have been examined in the same paradigm following forebrain (3V) and hindbrain (4V) administration. Our findings that highlight the effectiveness of acute hindbrain (4V) administration of OT to reduce short-term food intake are consistent with the recent findings from Ong and colleagues [24]. They found that acute 4V administration of slightly higher doses of OT (3, 6, and 12 μg/μL) suppressed food intake at 0.5 h post-injection while a lower dose (1 μg/μL) had no effect at this time point. It is not clear why lower doses (0.04–1 μg/μL) were effective at reducing food intake at 0.5 h post-injection in our hands. Both studies used Sprague-Dawley rats from the same vendor. However, differences with respect to paradigm (non-fasted [24] vs. 6-h fast in our study) may explain, in part, the increased sensitivity in our study. It is also important to note that two separate studies by both Ong [24] and Ho [36] have now demonstrated that the effects of acute 4V OT to reduce food intake, 1 μg [36] or 3 μg [24], were blocked in response to acute 4V administration of an OTR antagonist. These findings suggest that the effects of acute 4V OT to reduce short-term food intake are attributed to action at OTRs. Ong and colleagues further extended these findings and determined that injections of OT directly into the NTS, at a dose subthreshold for 4V effects in their study (1 μg), was effective at reducing 0.5-h food intake. They subsequently assessed the physiological role of NTS OTRs in the control of food intake and found that viral knockdown of NTS OTRs resulted in increased meal size and fasting-elicited refeeding [49]. These findings are consistent with earlier work by Baskin and colleagues, who found that NTS administration of OT-saporin toxin resulted in a modest stimulation of food intake in a rat model [50]. While the receptor populations that may contribute to the effects of acute 4V administration on short-term food intake were not identified in our studies, these findings raise the possibility that hindbrain NTS OTRs could be important in contributing to these effects.

Our observation that chronic hindbrain (4V) administration of OT reduces weight gain in HFD-fed CD^®^ IGS rats recapitulates our findings following 3V administration in CD^®^ IGS rats [29] and Long-Evans rats in the current study, suggesting that OT’s effects on hindbrain and/or spinal cord OTRs contribute to its effects on weight loss. Of note is the finding that 4V OT did not appear to elicit more sustained reductions of energy intake relative to what we had previously found following 3V administration in CD^®^ IGS rats [29] or in Long–Evans rats in the current study. Whether this is due, in part, to a more enhanced effect of OT to up-regulate its own expression and release when given into the 3V of CD^®^ IGS rats is not clear. OT can activate OT neurons, in part, through activation of OT auto-receptors on magnocellular SON [51,52] and PVN OT neurons [53]. Chronic lateral ventricular infusions of OT can up-regulate hypothalamic OT mRNA and increase OT levels within the circulation [27]. This has also been demonstrated for systemic OT, where it is known to increase Fos within PVN OT neurons [54,55,56] and stimulate the release of OT within the CNS [8]. These findings have been recently replicated in HFD-fed mice where peripheral administration of OT was found to increase Fos within PVN OT neurons [54] and up-regulate hypothalamic OT mRNA. Future studies will be required to determine the extent to which OTRs in specific hindbrain sites (i.e., NTS or raphe pallidus) are necessary for the anti-obesity effects of OT. Specifically, it will be important to address the extent to which site-specific ablation of OTRs in DIO rats can (1) block the ability of chronic 4V OT to reduce weight gain and adiposity and (2) predispose rats to the metabolic and behavioral abnormalities associated with diet-induced obesity (e.g., increased energy intake; increased visceral fat mass, body weight, and hyperlipidemia; reduced ambulatory activity and vascular dysfunction) [57,58].

Previous studies have suggested that OT inhibits food intake, in part, by enhancing the responsiveness to meal-related satiation signals, such as cholecystokinin (CCK). We and others have found that (1) central OTR antagonist administration lateral ventricle [59], hindbrain 4V [41], and (2) diptheria toxin-elicited reductions of PVN OT signaling [60] both attenuate the effectiveness of CCK-8 to reduce food intake. Furthermore, NTS administration of OT-saporin toxin (to lesion OTR expressing neurons within the NTS) was found to blunt the effects of CCK-8 to reduce food intake [50]. Recently, Ong and colleagues found that viral knockdown of NTS OTRs resulted in reduced sensitivity to gastrointestinal satiation signals elicited in response to a caloric preload [49]. Together, these findings suggest that endogenous OT action at hindbrain OTRs is able to enhance the effectiveness of gastrointestinal satiation signals (including CCK-8) to reduce food intake. In contrast, we found that chronic infusion of exogenous OT into the CNS (3V) was able to markedly enhance the effectiveness of lower doses of CCK-8 (0.25, 0.5 nmol/kg) to reduce HFD consumption [29]. In addition, Ong and colleagues found that NTS administration of OT, when given in combination with a nutrient preload, resulted in a greater suppression of food intake compared to either treatment alone [24]. Collectively, these findings support the hypothesis that increased OT signaling enhances sensitivity to meal-related satiation signals (including CCK-8) to reduce food intake.

While it is clear that CNS OT is important in the control of energy balance, we are only beginning to understand the downstream neurons that contribute to these effects. Although cNTS catecholamine neurons, some of which project to the PVN, are downstream targets of central (lateral ventricles [25], 4V) and peripheral OT action [36], what still remains unclear is whether cNTS catecholaminergic circuits may contribute to OT’s ability to suppress food intake. Previous anatomical findings showed that OT fibers that originate from the parvocellular PVN are found in close anatomical proximity to hindbrain NTS catecholamine neurons [40]. In addition, CCK-8-elicited activation of PVN OT neurons appears to occur through ascending noradrenergic (NA) innervation of the PVN from the A2 region of the cNTS [61,62] as lesions of cNTS NA neurons attenuate the effectiveness of CCK-8 to activate PVN OT neurons [63]. Furthermore, alpha-1 adrenoceptor activation in the PVN is associated with reductions in food intake [64,65], and alpha-1 adrenoceptor 1D mRNA is expressed on PVN OT and corticotrophin-releasing hormone (CRH) neurons [66]. Based on these collective findings and previous findings, we hypothesized that OT may suppress food intake, in part, by enhancing the effectiveness of CCK-8 to activate cNTS catecholamine neurons that project to PVN. However, we did not find that systemic administration of prazosin, a drug that blocks alpha-1 adrenoceptors (at a dose that blocked the effects of the alpha-1 adrenoceptor agonist, cirazoline, to inhibit food intake), impacted the ability of systemic OT to suppress food intake [36], suggesting that systemic OT does not reduce food intake through an alpha-1 adrenoceptor-dependent mechanism. Future studies will be needed to more definitively rule out the involvement of the alpha-1 adrenoceptors in contributing to the effects of central OT-mediated reductions of food intake. It is possible that other noradrenergic receptor types may be important or that alpha-1 adrenoceptors in HFD-fed animals may function as heterodimers with other receptors to contribute to the ability of OT to reduce food intake.

NTS GLP-1 and POMC neurons have also been identified as potential downstream targets of CNS OT. NTS OT fibers are found in anatomical proximity to NTS GLP-1 neurons, and lateral intracerebroventricular (ICV) administration of OT induces Fos in NTS GLP-1 neurons [25]. Furthermore, intracerebroventricular (lateral) administration of the GLP-1 receptor (GLP-1R) antagonist (des His1 Glu9-exendin 4) attenuates the ability of ICV OT to reduce chow intake [25]. NTS OT terminals are also in anatomical proximity to NTS POMC neurons and OT evokes the release of intracellular Ca^2+^ from NTS POMC neurons [15]. Furthermore, 3V administration of the melanocortin 3/4 receptor antagonist, SHU9119, blocks the effects of 3V OT to inhibit chow intake [15]. It will be important to assess the extent to which these neuronal populations are also downstream targets of 4V OT action, given that these earlier studies examined the effects of OT on activation of GLP-1 or POMC neurons following a forebrain route of administration (lateral ventricles, 3V; [15,25]).

In summary, we found that both 3V and 4V administration of OT can effectively reduce food intake in chow-fed lean rats, as well as both food intake and weight gain in HFD-fed rats. Acute administration of OT into the 3V and 4V OT (5 μg) suppressed 0.5-h food intake to a comparable extent. The 4V OT at lower doses (0.04, 0.2, 1 μg) reduced short-term food intake in a dose-dependent manner, whereas 3V administration of OT at only one of the lower doses (1 μg) was effective at reducing short-term food intake. Similar to what we observed at the high dose, there were comparable effects in response to 3V or 4V administration at the lower doses. When given chronically into the 3V and 4V of HFD-fed rats, OT reduced weight gain and adiposity. These effects were accompanied by transient reductions of energy intake. Lastly, we found that 4V OT increased the number of cNTS Fos(+) neurons and induced Fos in TH (+) neurons relative to vehicle. Collectively, these findings support the hypothesis that OT within the hindbrain is effective at reducing food intake, weight gain, and adiposity and that NTS catecholamine neurons in addition to non-catecholaminergic neurons are downstream targets of CNS OT. It will be important to address the extent to which ablation of NTS OTRs may impact the ability of 4V administration of OT to reduce food intake, body weight, and adiposity in future studies. 

## Figures and Tables

**Table 1:** Plasma measurements following 4V infusions of OT or vehicle in male HFD-fed CD^®^ IGS rats.

4V Treatment	Vehicle	OT
	HFD	HFD
**Leptin (ng/mL)**	25.4 ± 4.5 ^a^	16.2 ± 2.6 ^a^
**Insulin (ng/mL)**	2.1 ± 0.5 ^a^	2.0 ± 0.4 ^a^
**Adiponectin (µg/mL)**	5.7 ± 0.3 ^a^	5.1 ± 0.4 ^a^
**Blood Glucose (mg/dL)**	138 ± 3.1 ^a^	144 ± 5.5 ^a^
**FGF-21 (pg/mL)**	150 ± 34 ^a^	124 ± 21.2 ^a^
**Irisin (mg/mL)**	6.6 ± 0.7 ^a^	6.8 ± 0.8 ^a^
**FFA (mEq/L)**	0.2 ± 0.05 ^a^	0.1 ± 0.01 ^a^
**Total Cholesterol (mg/dL)**	67.4 ± 4.3 ^a^	70.8 ± 3.3 ^a^

Data are expressed as mean ± SEM. Different superscript letters denote significant differences between treatments. Shared letters are not significantly different from one another. FFA, free fatty acids; FGF, fibroblast growth factor-21; HFD, high fat diet; OT, oxytocin. Leptin (Vehicle vs OT (*P* = 0.1)). N = 7–8/group.

**Table 2:** Plasma measurements following (A) chronic 4V infusions of OT or vehicle or (B) washout period in male DIO CD IGS rats.

Table 2A. Plasma Measurements Following 4V Infusions of Oxytocin or Vehicle in DIO CD IGS Rats				
4V Treatment	Vehicle	OT	Vehicle	OT
	CHOW	CHOW	HFD	HFD
**Leptin (ng/mL)**	10.1 ± 1.2 ^c^	12.3 ± 1.7 ^c^	39.4 ± 4.2 ^a^	27.6 ± 5.8 ^b^
**Insulin (ng/mL)**	0.8 ± 0.09 ^a^	1.3 ± 0.2 ^ac^	1.4 ± 0.4 ^bc^	0.7 ± 0.05 ^ac^
**Adiponectin (µg/mL)**	5.0 ± 0.2 ^b^	5.4 ± 0.3 ^b^	6.9 ± 0.5 ^a^	6.8 ± 0.6 ^a^
**FGF-21 (pg/mL)**	71.8 ± 11.3 ^b^	83.6 ± 19.9 ^cb^	138.2 ± 25.9 ^ac^	161.9 ± 15.5 ^a^
**Irisin (mg/mL)**	11.7 ± 0.7 ^a^	11.3 ± 0.7 ^a^	11.0 ± 0.3 ^a^	10.5 ± 0.7 ^a^
**FFA (mEq/L)**	0.18 ± 0.03 ^b^	0.2 ± 0.04 ^b^	0.3 ± 0.05 ^a^	0.2 ± 0.03 ^ab^
**Total Cholesterol (mg/dL)**	55.4 ± 1.7 ^b^	53.8 ± 4.5 ^b^	71 ± 9.4 ^a^	65.9 ± 8.1 ^ab^
**Table 2B. Plasma Measurements at Washout**				
	**VEH Washout**	**OT Washout**		
	**HFD**	**HFD**		
**Leptin (ng/mL)**	40.4 ± 3.7 ^a^	30.7 ± 4.8 ^a^		
**Insulin (ng/mL)**	1.3 ± 0.3 ^a^	1.7 ± 0.3 ^a^		
**Adiponectin (µg/mL)**	7.1 ± 1.0 ^a^	6.8 ± 0.5 ^a^		
**FGF-21 (pg/mL)**	141.9 ± 37.9 ^a^	102.1 ± 13.6 ^a^		
**Irisin (mg/mL)**	12.8 ± 1.2 ^a^	11.6 ± 1.2 ^a^		
**FFA (mEq/L)**	0.22 ± 0.03 ^a^	0.19 ± 0.03 ^a^		
**Total Cholesterol (mg/dL)**	78.6 ± 9.6 ^a^	67.8 ± 4.9 ^a^		

Data are expressed as mean ± SEM. OT vs. vehicle. Different superscript letters denote significant differences between treatments. Shared letters are not significantly different from one another. FFA, free fatty acids; FGF, fibroblast growth factor-21; HFD, high fat diet; OT, oxytocin. (A) N = 4–11/group. (B) N = 5–6/group.

## Data Availability

The data will be made available upon written request and when published, through the online journal itself as well as through U.S. National Library of Medicine at the National Institutes of Health (PubMed) and similar sites.
